# Typhus or Ship-Fever at Bellevue Hospital, New York

**Published:** 1847-11

**Authors:** B. F. Wendel

**Affiliations:** One of the Assistant Physicians; Bellevue Hospital


ART. II.—*Typhus or Ship-Fever at Bellevue Hospital, New York.* By B. F. WENDEL, M.D., one of the Assistant Physicians.

This disease, as occurring here, is intimately associated with immigration and famine, most of our cases being presented in the persons of those recently arrived from starving countries. You have but a faint conception of the actual state of the paupers arriving in this city. Many of the immigrants have assured me that during the voyage they went without bread for several days, and without water for as long a time; and that often half a dozen men, women and children were crowded into a room scarcely large enough for one. But their looks bespeak the condition in which thev have lived. Those who are not attacked on shipboard, being mostly poor people, on arriving go into the most confined and crowded parts of the city, where they run into all manner of excesses, and soon the latent disease is developed in them.

*Symptoms.*—Most of our patients are brought to the hospital after the disease is fully developed, but in those who come in early, or before the attack, the premonitory symptoms are in some cases quite severe. Sometimes a day or two previous to the attack there is nothing more than a general *malaise*, an inaptness for everything either physical or mental, weakness and wandering pains in the limbs and head, and more or less throughout the body, a deficiency in the secretions of the skin and kidneys, and a sluggish state of the bowels. In other instances the attack is exceedingly sudden, (in one case which fell under my charge, the attack was so sudden, and the headache so severe, that the patient's friends took it to be a sunstroke,) being preceded by an intense headache. In other cases, nausea and vomiting usher in the disease.

Such are some of the modes of attack. For the first few days, in such cases as the above, the tongue is pretty heavily coated, and but slightly moist; the skin is hot and dry, and the pulse full, frequent and compressible. Added to these is a dull and heavy pain in the head, or, as the patients express it, a sensation as if the head would burst; and just in this condition it is that obstinate epistaxis is apt to occur. Soon this stage passes into that in which most of our patients arrive at the hospital.

The skin now becomes dusky, the eyes congested and protruding, or seemingly insensible, the pain in the head abates or continues, and a wild delirium, or one of a low and muttering character, comes on; the patient lies unconscious of what is passing around him; the tongue is like a piece of crisped meat; in many cases there is inability to void urine, the secretion of which is sometimes suspended. In one case, although the man had not made water for a day and a half, on introducing the catheter, the bladder was found almost empty. The bowels are either costive or the reverse, one condition being about as frequent as the other; the pulse is feeble and frequent, and the patient lies nearly unconscious and unable to give an intelligible answer to your questions.

In this stage also the hearing is greatly affected, so much so that you cannot make the patient understand what you say; and with it there is inability to protrude the tongue. As this stage progresses, the tongue remains dry and parched, the skin seems scaly, the breathing becomes more hurried, the sordes increase, and the pulse grows more feeble and irregular, until death closes the scene. But should the disease take a favorable turn, it is indicated by a return of moisture to the skin and tongue, a gradual disappearance of the sordes from the teeth, and a pulse of more force and regularity.

*Complications and Sequences.*—Diarrhea and dysentery either complicate or follow the fever, but more frequently the latter is the case. Parotitis and suppuration of the parotid is not uncommon, both during the course of and succeeding to the disease. Œdema of the lower extremities follows most of the cases of fever, and often the feet and legs are enormously swollen. Erysipelas is common after fever, generally affecting the scalp and face, though it is apt to attack any abraded surface.

*Pathological Appearances.*—In the autopsy of some twenty-five cases which fell under my notice, the organs chiefly affected were, in the order of frequency, the *encephalon*, the *spleen*, and the *lungs*. In all these cases there was subarachnoidal effusion, and effusion at the base of the brain and into the ventricles. The substance of the brain was sometimes slightly softened. The spleen was generally softened, and often so much so as to fall in pieces. It was not at all uncommon to find the lungs in a state of congestion.

*Treatment.*—When a case is seen in its incipiency, and the patient presents a furred tongue, hot and dry skin, with a full and compressible pulse, generally the first thing is to clear the bowels by a gentle cathartic; then give small doses of ipecacuanha, or pulvis antimonialis compositus, until these symptoms are somewhat reduced, and afterwards administer acetate of ammonia. But in the condition in which most of the patients arrive, this practice is not required. The course usually pursued is to evacuate the bowels, if necessary, by a dose of oil, or an injection of soap and water, and then give a dose of spiritus mindereri every hour or two according to circumstances; meantime sponging the surface with cool or tepid water, once or twice a day, and letting the patient eat plenty of ice. Local symptoms are sometimes relieved by cups and leeches, though these are scarcely ever called for. Where there is much headache, solid ice or cloths wrung out of cold water are applied to the head. If symptoms of sinking come on, the patient is supported by carbonate of ammonia and brandy, milk punch, wine whey, etc. As to diet, it consists of rice-water, barley-water, oatmeal gruel, beef tea, etc. The treatment of this disease is very simple, and is directed chiefly to the support of the vital powers.

I close my brief communication with the report of a few cases illustrating some of the sequelæ of ship fever:

Mary B., aged eighteen years, of nervous temperament and weak constitution, was just recovering from fever, which had left her much debilitated, and with a great tendency to diarrhea. She was attacked with a slight chill on the evening of the 9th of September, which left her with pain in the head, a hot and dry skin, and a little dyspnœa. I saw her on the morning of the 9th, and found her with a frequent and feeble pulse, a tongue red and dry, eyes congested, irritability of the stomach and bowels, pain about the middle of the right side of the chest, but little expectoration, and that a thick matter tinged very slightly with blood, and mingled with froth. Percussion gave no clue to the state of the chest. Auscultation revealed minute crepitation, which was confined to the right lung and to its middle portion, mingling more and more with the vesicular murmur as you ascended or descended the chest; the left lung gave puerile respiration and a slight mucous rale.

Treatment: ℞. Rad. seneca, rad. scillæ, of each ʒj, and a small stick of licorice, on which pour half a pint of water, and, after cooling, take a table-spoonful every three hours.

10th. Does not feel well; spent a restless night; pulse still frequent and feeble; breathes with a little more ease; eyes congested; some headache; tongue red but moist; much tendency to diarrhea. The characteristic sputa appeared today. Sounds of the chest about the same as yesterday.

Ordered to continue the former prescription, in the same quantity as yesterday, every two hours, and ten grains of Dover's powder to be taken at bedtime.

11th. Rested well; skin moist and cool; pulse not so frequent, but feeble; tongue moist and red; less pain in the head and chest, and bowels very loose. Crepitation and puerile respiration are not so distinctly audible as yesterday.

Continue the first prescription, and take a teaspoonful of a mixture composed of tinct. of catechu ʒij, camphorated tincture of opium ℥j, and prepared chalk ℥j, after every other stool; and ten grains of Dover's powder at bedtime.

12th. Improving; slept well; pulse of more regularity; eyes a little congested, but no more than is natural with her; head feels heavy, but is not painful, and the pain in the side is gone; the expectoration is free, and the cough soft; crepitation and puerile respiration much diminished.

Continue the first prescription and the diarrhea mixture.

13th. Feels very well, though quite weak; all the signs of pneumonia had nearly vanished, and the bowels were in check.

Ordered to continue the first prescription, in the same quantity, every six hours, together with the astringent mixture.

14th. Convalescent; only complains of weakness.

A woman aged twenty-four years, who enjoys generally good health—whom I had attended during an attack of ship-fever about two months since, and also during an abortion at the seventh month—was suddenly seized with a chill and great difficulty of breathing. The symptoms grew more and more urgent; and in about twenty minutes after the cessation of the chill, the nurse ran into my room, exclaiming that the woman was dying. On getting to the ward, I found her in the following condition: She was sitting up in bed, her countenance extremely anxious, and her lips purple; she was gasping for breath, making about fifty respirations in a minute; the pulse was one hundred and thirty, feeble and quick; her skin covered with cold perspiration; the tongue pale, and covered with a brownish white fur; she had most intense pain running from the false ribs of the left side upwards and forwards to the sternum. There was no expectoration.

*Physical Signs.*—Percussion revealed the most intense dullness at the lower part of the left lung, with a diminution of the healthy resonance over the other portions of it. Auscultation gave a distinct crepitation, which was audible over the lower lobe of the left lung and over some portion of the upper, with some friction sound. The right lung gave most intense puerile respiration.

*Treatment.*—Bled from the arm fifteen ounces, with a very marked relief of the symptoms, (the blood was buffed;) gave her ten grains of calomel with five of rhubarb, and ordered two grains of tartar emetic to be dissolved in two ounces of water, of which take a teaspoonful every half hour.

Morning. Bowels had been moved freely; skin moist and pleasant; pulse one hundred, and of some force; no pain any where; tongue moist.

Percussion gave but little less than the healthy resonance on the left side, and auscultation afforded but little more than minute crepitation mingling, to a slight extent, with the continuous murmur in the same lung, and in the right lung very slight puerile respiration.

Ordered to continue the tartar emetic.

Evening. In about the same condition as this morning.

Ordered to continue the tartar emetic in the same dose every hour during the night, ten grains of Dover's powder to be taken at bedtime, and a dose of oil in the morning.

Morning. In still better condition; slept well; bowels moved freely; pulse of more force and less frequency; skin pleasant; she feels very well. Percussion gave no dullness, and auscultation showed the vesicular murmur to be fast replacing the crepitant rale.

Ordered to continue the tartar emetic every two hours.

Evening. Still improving; says she intends to get up in the morning.—Ordered a dose of oil for the morning.

Morning. Found her walking about the room; told me she felt as well as she ever did.

*Remarks.*—The disease was situated in the left lung in this instance. I at first thought that there must be great effusion into the pleura on the left side, but change of position did not justify me in the belief, nor did the rapid recovery. The characteristic sputa of pneumonia was wanting.

Carr, an inmate of the almshouse, aged seventy-six years, who had generally enjoyed good health, save now and then being subject to palpitations of the heart and occasional attacks of asthma, was suddenly seized with great pain in the hypogastric region, and inability to urinate, about four o'clock in the afternoon of the 9th of September. Applications were made which soothed the pain, but without enabling him to make water.

The following morning, although he had not made water in twenty-four hours, there was but little distension in the region of the bladder. The catheter was introduced, and about five ounces of water escaped, which was followed by an alleviation of the pain.

His bowels had not now been moved in two days, and in the afternoon of this day he began to complain of great pain over the abdomen, and "cutting in the bowels," which was relieved by pressure. Four compound cathartic pills were administered at bedtime, and were followed by a dose of oil next morning, but without producing the desired effect; his water in the mean time being drawn off by the catheter. The oil was followed by mild and powerful injections, and by the most potent cathartics, but all without avail. During the evening of this day he had an attack of asthma, and next day, about eleven o'clock, died.

The autopsy showed that a most intense peritonitis had been going on. The bowels were so firmly agglutinated that it was nearly impossible to separate them; and over and above these old adhesions there was a large quantity of recent lymph, and the peritoneum lining the abdomen was covered with the same. The lungs were in an emphysematous condition; the other organs were healthy.

I mention this case to show that pain on pressure is not invariably present in peritonitis, of which this is a good example, for this man devised all manner of means to increase the pressure on his abdomen.

Bellevue Hospital, September 20, 1847.

